# Regression of metastatic malignant melanoma with dupilumab: A case report

**DOI:** 10.1002/ski2.362

**Published:** 2024-03-14

**Authors:** John Herbert Marr, Abbas Al‐Shammari

**Affiliations:** ^1^ Consultant Medical Advisor Suffolk UK; ^2^ Department of Dermatology Locum Consultant Dermatologist West Suffolk Hospital Suffolk UK

## Abstract

Excoriated pruritus can be an intolerable symptom in patients with cancer where Type 2 inflammation and its associated cytokines IL‐4 and IL‐13 play major roles in the pruritus. Dupilumab, an antibody blocking IL‐4 and IL‐13, is approved for treating moderate to severe atopic dermatitis (AD) where itching is a significant symptom. We present a case report of intractable malignancy‐associated AD and pruritus with eosinophilia in a patient with stage IV malignant melanoma who was treated with dupilumab. Biweekly treatment with dupilumab led to an immediate improvement in itching and resolution of the AD, which subsided after a few doses and without significant adverse effects. Routine radiologic monitoring of the malignant melanoma showed concomitant resolution of secondary nodules in the lung, liver, and pleura. It was concluded that dupilumab may be a safe and effective treatment for intractable malignancy‐associated AD with pruritus and may have potential for moderating metastatic malignant melanoma.

## Graphical abstract

This is a case report of regression of metastatic malignant melanoma with dupilumab. The production of cytokines by tumour cells has significant effects on cancer cell progression and metastasis, and increased expression of Interleukin‐4 (IL‐4) and IL‐13 by immune and non‐immune cells in the tumour microenvironment result in activation of tumour growth, survival, and immunosuppression. Dupilumab may be used to manage neoplastic dermatitis. It blocks IL‐4 and IL‐13 to disrupt signalling and may therefore also modulate tumour progression. Further studies and case reports will support the safety profile of dupilumab in managing malignancy‐associated atopic dermatitis. Additionally, dupilumab may moderate tumour progression and metastasis in malignant melanoma.

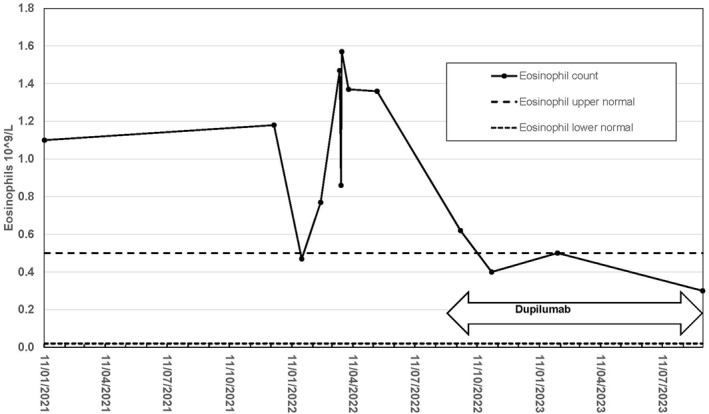

## 1 INTRODUCTION

Malignant melanoma is a highly aggressive, heterogeneous tumour accounting for about 1%–4% of all skin cancers globally but 65% of skin cancer deaths,^1^ with a strong tendency to metastasise to other organs.^2^, ^3^ Intermittent sun exposure, a family history, and prior removal of melanomas are significant risk factors,^1^, ^3^, ^4^ with the highest incidence and mortality in Australia and New Zealand, followed by North America and Northern and Western Europe. The continuous increase in melanoma prevalence has become a major clinical problem with an average yearly increase of more than 4% globally,^5^ although the incidence has remained steady over the last few years.^6^ In early‐stage melanoma, surgical removal of the tumour has a curable outcome in around 90% of patients. For patients with disseminated disease however, the outcomes are still poor despite the recent emergence of immune checkpoint inhibitors and targeted treatments.^7^

We present the case of a 75‐year‐old male diagnosed with BRAF negative stage IV malignant melanoma who developed flare‐up of AD responsive to dupilumab. Radiologically confirmed metastases to the lung, liver and pleura had cleared 1 year after commencement of dupilumab.

## 2 CASE REPORT

A 75‐year‐old male, diagnosed with Breslow 1.2 mm melanoma of the left pinna (pT2a) in 2017 was treated with wide local excision plus sentinel lymph node biopsy (WLE + sentinel lymph node biopsy), both negative. CT Chest in 2018 indicated bilateral upper lobe scarring and mild right upper lobe bronchiectasis possibly due to previous TB. Posteriorly, there was a subpleural well defined nodule in the right lower lobe, partially triangular in outline and in keeping with a subpleural lymph node.

In October 2018 he developed muscle‐invasive bladder cancer approximately 3 cm across, confirmed histologically after trans‐urethral resection as pT2G3 high grade urothelial transitional cell carcinoma, with muscularis propria invasion, but no extra‐vesicular spread. This was treated with 20 sessions of radical chemoradiotherapy to the bladder with Bladder carbogen and nicotinamide radiotherapy protocol. Routine check cystoscopies continued to show no recurrence.

Surveillance scans for malignant melanoma disclosed an enlarging lung nodule. CT scan in July 2020 showed bilateral apical pleural scarring, scarring atelectasis in the right upper lobe with bronchial wall thickening bilaterally, and a soft‐tissue nodule of 3.5 mm in left lower lobe superior segment, grossly unchanged since September 2018 and December 2019. High‐resolution computed tomography in July 2021 showed the nodule increasing in size to 9 mm. Further CT follow‐up at 2 months in September 2021 showed further increase in the lung metastasis to 11 mm, with no evidence of other recurrent disease with magnetic nuclear resonance. FDG‐avid positron emission tomography scan showed this as solitary disease, and biopsy of the apical segment of the left lower lobe followed by left lower lobe wedge resection confirmed oligometastatic melanoma with clear margins (R0 resection). Adjuvant nivolumab immunotherapy was commenced in December 2021, but was interrupted after three sessions following febrile acute renal failure with diarrhoea and nausea.

CT in April 2022 showed right lower lobe (RLL) subpleural nodule of 6 mm, and left apical nodule with minimal right pleural effusion, a solitary liver node and other scattered small attenuating lesions in the liver presumed benign. Repeat CT in August 2022 showed multiple small‐volume hilar, mediastinal and axillary lymph nodes increasing in size, right hilar lymph node now 10 mm, right pulmonary nodule increased from previous scan to 10 mm, and increased apical thickening. The low‐attenuating node in the liver had increased to 14 mm from previous 4 mm, with the radiological conclusion of progressive disease.

Concurrently, the patient had increasingly severe flare‐up of malignancy‐associated generalised AD with severe pruritus and excoriation which had become progressively unresponsive to standard topical therapy, including potent dermal steroids and short courses of prednisolone. Ultraviolet therapy was contraindicated with the history of melanoma. In August 2022, he was therefore commenced on dupilumab with an initial dose of 600 mg S.C. followed by 300 mg every 2 weeks. Three months later, following resolution of the multiple areas of AD and associated symptoms, he was able to taper off prednisolone and could stop all topical steroids, emollients, and antihistamines, and currently remains symptom free.

**FIGURE 1:**
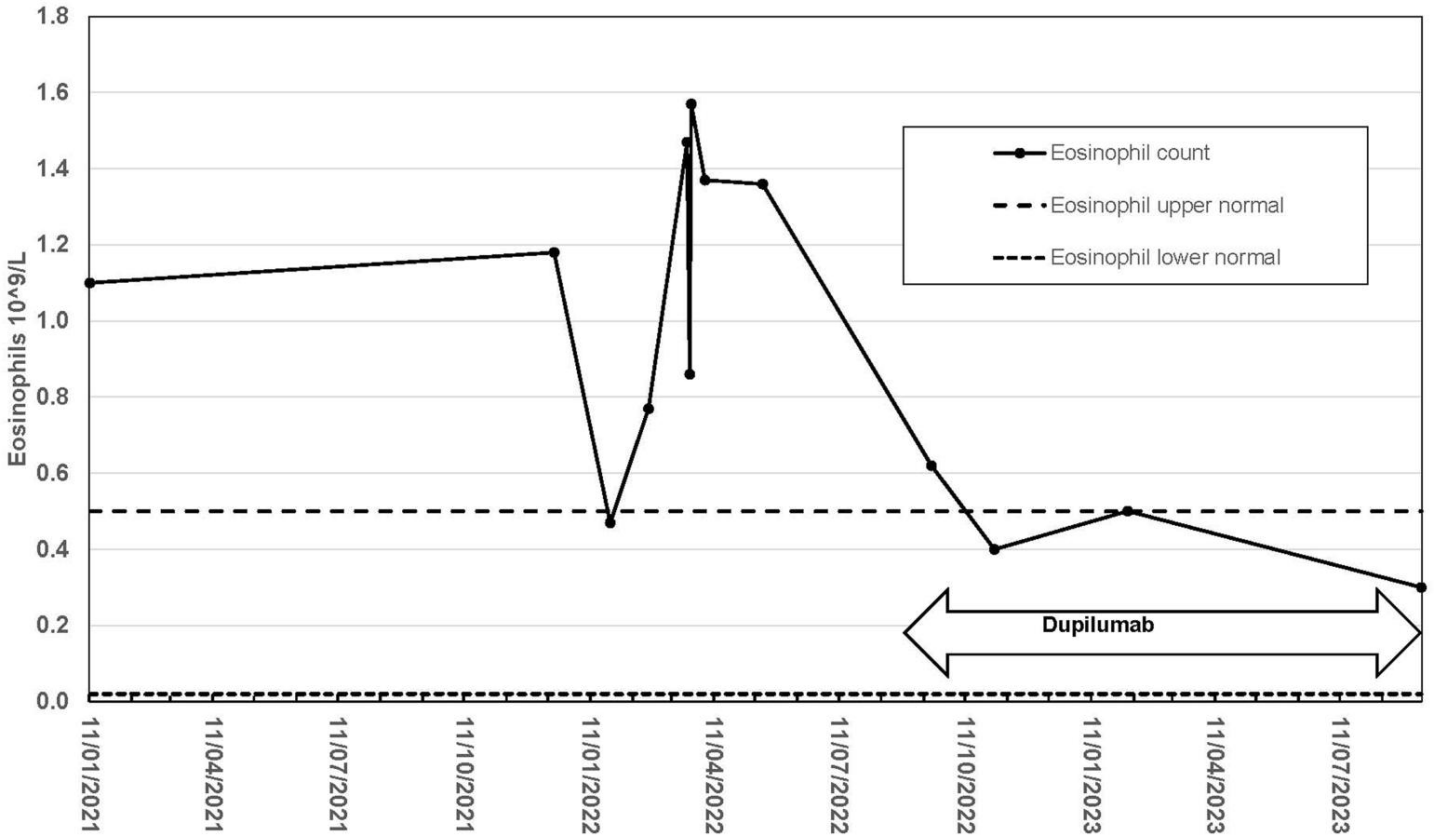
Eosinophilia normalisation in malignant melanoma with dupilumab.

A repeat scan In January 2023 showed interval reduction in the RLL nodule from 10 to 8 mm, interval reduction in size of previous enlarged lymph nodes with no pleural effusion, and interval reduction in the liver nodule from 12 to 7 mm. Eosinophilia was recorded over much of this period (Figure 1), increasing from 1.0 to 1.6 × 10^9^/L (normal range 0.02 − 0.50 × 10^9^/L), but falling within normal limits with the commencement of dupilumab. A corresponding lymphopenia was noted over the same period, again returning to normal with dupilumab. Repeat interval contrast CT of head, neck, chest, abdomen, and pelvis in September 2023 confirmed no lymphadenopathy, with no liver metastasis identified. The subpleural RLL nodule was unchanged since 2021. There was radiological and symptomatic evidence of acute calculus cholecystitis, but no evidence of metastatic disease was identified. Clinically he remained in good general health with no recurrence of AD.

## 3 DISCUSSION

Atopic dermatitis brings a great burden to patients. Pruritus is a key feature, with pruritus‐excoriated xerosis and lichenification leading to a cycle of increasing pruritus. Moisturising ointments, topical steroids, calcineurin inhibitors, and other conventional therapies are of limited use, especially in moderate to severe AD. Dupilumab is the first monoclonal antibody approved for treatment of AD.^8^ It is a humanised IgG4 antibody with key roles in Type 2 inflammatory responses triggered by allergens and mediated by T helper 2 (Th2) cells and blocks the structurally similar cytokines Interleukin‐4 (IL‐4) and IL‐13 to disrupt signalling through the type I and type II receptor complexes.^9^, ^10^ Clinical trials for dupilumab did not include patients with cancer, and current immunosuppressive agents used for the treatment of AD raise safety concerns when being used for patients with malignancies with the possibility of exacerbation of cancer, although several recent reports have found no evidence linking dupilumab with cancer recurrence.^11^, ^12^, ^13^

There are many components to the tumour microenvironment (TME) in epithelial cancers, including fibroblasts, immune and inflammatory cells, blood and lymph vessels, and nerves, all with the potential to influence tumour behaviour. Tumour‐associated macrophages are particularly plentiful at all stages of tumour growth, and these have been shown to facilitate angiogenic responses and promote tumour proliferation.^14^ Immune cell infiltration into solid tumours, their movement within the TME, and their interaction with other immune cells, are controlled by their directed migration towards gradients of chemokines, and dysregulated chemokine signalling in TME favours the growth of tumours, exclusion of effector immune cells, and abundance of immunosuppressive cells.^14^

The production of cytokines by tumour cells has been directly linked to aggressive tumour growth, invasion, metastasis, and the suppression of tumour‐directed immune surveillance mechanisms, and rather than diminishing inflammation and helping to eradicate tumour cells, both IL‐4 and IL‐13 have shown significant effects on cancer cell survival, progression and metastasis.^15^ Specifically, IL‐13 has recently been demonstrated to repress tumour surveillance and inhibit tumour rejection, with increased expression of IL‐4, IL‐13, and their receptors by immune and non‐immune cells in the TME resulting in activation of tumour growth, survival, and immunosuppression.^10^

This over‐expression of IL‐4/IL‐13 and their receptors in certain cancers, combined with their stimulative roles for tumour progression and their ability to bind to their receptors with high efficacy and specificity, has led to several clinical studies to assess the safety and efficiency of drugs targeting these cytokines in several diseases,^15^, ^16^, ^17^, ^18^ including pancreatic cancer,^15^ multiple myeloma^18^ and relapsed/refractory metastatic non‐small cell lung cancer.^19^ Furthermore, a strong link is emerging between the over‐expression of IL‐4/IL‐13 and the role of eosinophils in the immune reaction to invasive cancers.

Eosinophils are a minor population of granulocytes that are mostly considered in asthma and allergic disorders, where they are polarised in the presence of Th1 or Th2 cytokines. Eosinophils generally circulate for 1–2 days before elimination, but some chemokines significantly increase their lifespan and attract them to migrate into inflamed tissues and certain tumours, including the site of melanoma growth.^20^ They also secrete several chemokines that entice natural killer cells, activated macrophages, and CD8+ T‐cells to proliferating tumour cells.^21^ Additionally, Th2 cells have been reported to clear lung metastases in CTL‐resistant melanoma through eosinophil tumour infiltration and degranulation, and to hinder tumour progression by enhancing eosinophils.^22^ Peripheral blood eosinophilia prior to immunotherapy has been observed in patients who present with late stage 4 or widely metastatic melanoma,^23^ and the number of eosinophils in lymph nodes with melanoma metastasis has been demonstrated to be significantly lower, with more CD4+ cells than the normal lymph node.^20^

Expression of the chemokine receptor CXCR4 is known to regulate melanoma metastasis to distant sites, and CXCR4/CXCL12 play an important role in chemotaxis, cell growth, and cell migration of eosinophils. High IL‐4 with decreased CXCR4 on eosinophils induces dysregulation of migration, chemotaxis, and homing of eosinophils, and Ryser et al.^24^ have shown that CXCR4 is significantly down‐ and IL‐4 up‐regulated in subjects with eosinophil increase, with elevated IL‐4 values and decreased CXCR4 expression on eosinophils in patients with melanoma.

The role of eosinophilia in peripheral blood and metastatic tissue of cutaneous melanoma has been controversial; eosinophils express PD‐1 and its ligand PD‐L1, but the anti‐ or pro‐tumourigenic role of those eosinophils is cancer‐specific and may have opposite effects in various types of metastatic tumours through their effect on other immune cells to promote or suppress tumour growth.^25^ However, the underlying immune dysfunction in some human diseases can be ascribed to a lack of appropriate balance between Th1, Th2, and Th17 immune responses, including allergic inflammation, fibrosis, with some autoimmune diseases and cancers showing a predominance of Th2 immunity.^10^ Further studies to examine a potential role and mechanism for dupilumab in modulating metastatic malignant melanoma may therefore be justified.

## CONFLICT OF INTEREST STATEMENT

The authors declare no conflicts of interest.

## AUTHOR CONTRIBUTIONS

**John Herbert Marr**: Conceptualization (lead); Writing – original draft (lead); Writing – review & editing (equal). **Abbas Al‐Shammari**: Conceptualization (supporting); Writing – original draft (supporting); Writing – review & editing (equal).

## ETHICS STATEMENT

Ethical review and approval were waived for this report due to no patient involvement.

## Data Availability

Restrictions apply to the availability of these data. Original data is held by the radiography departments and are available from the authors with the permission of the radiography departments.
